# Identifying urban built environment factors in pregnancy care and maternal mental health outcomes

**DOI:** 10.1186/s12884-021-04056-1

**Published:** 2021-09-04

**Authors:** Yiye Zhang, Mohammad Tayarani, Shuojia Wang, Yifan Liu, Mohit Sharma, Rochelle Joly, Arindam RoyChoudhury, Alison Hermann, Oliver H. Gao, Jyotishman Pathak

**Affiliations:** 1 Department of Population Health Sciences, Weill Cornell Medicine, 425 East 61st Street, NY New York, USA; 2 Department of Emergency Medicine, Weill Cornell Medicine, New York, NY USA; 3 School of Civil and Environmental Engineering, Cornell University, Ithaca, NY USA; 4 Tencent Jarvis Lab, Shenzhen Guangdong, China; 5 Department of Obstetrics and Gynecology, Weill Cornell Medicine, New York, NY USA; 6 Department of Psychiatry, Weill Cornell Medicine, New York, NY USA

**Keywords:** Pregnancy care, Postpartum depression, Built environment

## Abstract

**Backgrounds:**

Risk factors related to the built environment have been associated with women’s mental health and preventive care. This study sought to identify built environment factors that are associated with variations in prenatal care and subsequent pregnancy-related outcomes in an urban setting.

**Methods:**

In a retrospective observational study, we characterized the types and frequency of prenatal care events that are associated with the various built environment factors of the patients’ residing neighborhoods. In comparison to women living in higher-quality built environments, we hypothesize that women who reside in lower-quality built environments experience different patterns of clinical events that may increase the risk for adverse outcomes. Using machine learning, we performed pattern detection to characterize the variability in prenatal care concerning encounter types, clinical problems, and medication prescriptions. Structural equation modeling was used to test the associations among built environment, prenatal care variation, and pregnancy outcome. The main outcome is postpartum depression (PPD) diagnosis within 1 year following childbirth. The exposures were the quality of the built environment in the patients’ residing neighborhoods. Electronic health records (EHR) data of pregnant women (*n* = 8,949) who had live delivery at an urban academic medical center from 2015 to 2017 were included in the study.

**Results:**

We discovered prenatal care patterns that were summarized into three common types. Women who experienced the prenatal care pattern with the highest rates of PPD were more likely to reside in neighborhoods with homogeneous land use, lower walkability, lower air pollutant concentration, and lower retail floor ratios after adjusting for age, neighborhood average education level, marital status, and income inequality.

**Conclusions:**

In an urban setting, multi-purpose and walkable communities were found to be associated with a lower risk of PPD. Findings may inform urban design policies and provide awareness for care providers on the association of patients’ residing neighborhoods and healthy pregnancy.

**Supplementary Information:**

The online version contains supplementary material available at 10.1186/s12884-021-04056-1.

## Background

The built environment, referring to the surroundings and physical artifacts of where humans live, is considered to be one of the five major social determinants of health (SDoH) [1]. The built environment is strongly associated with our way of life through determining the housing quality, mode of transportation, and exposure to pollutants, among others. Poor built environment has been reported to lead to adverse effects on physical and mental health by disrupting sleep, hindering healthy lifestyles, and lowering access to healthcare [2–5]. There is a gender difference in the association between the built environment and health. For example, an increased risk of depression among female was reported by Mulling et al. to be associated with living in an underdeveloped neighborhood characterized by inadequate sewer treatment, water supply, and dependable supply of electricity [6]. In addition, the Chicago Community Adult Health Study found the women’s use of preventive care to be associated with objective and perceived neighborhood support and stressors such as odors, presence of trees, and noise levels [7].

The existing literature motivated this study to examine the impact of the built environment on health and healthcare utilization among women, and particularly, pregnant women as a population to further investigate [8–10]. Levels of prenatal care vary across the United States [11–13]. A substantial proportion of pregnant women, especially those with a higher comorbidity burden or low health literacy, seek and depend on care provided by emergency departments (ED) rather than primary and obstetric care [13–15]. The lack of adequate prenatal care is considered a risk factor for poor pregnancy outcomes and lack of proper postpartum care for mothers and infants [16]. Previous studies have studied the built environment on maternal health and birth outcomes including birth weight, gestational age, Apgar score, and newborn intensive care unit admission rates [5, 17]. Yet, evidence is still accumulating on how the built environment affects the variability in prenatal care and maternal mental health outcomes. In particular, few studied the concurrent impacts of prenatal care and built environment on mental health outcomes [18–22]. Existing studies have also commonly relied on the subjective perceived measures obtained from interviews and questionnaires to define postpartum depression (PPD), thus limiting larger-scale analysis [7, 18, 19, 23].

A study conducted in Mexico found that an increase in average particulate matter ≤ 2.5 μm in diameter (PM_2.5_) exposure during pregnancy was statistically associated with an increased risk of PPD at six months and also for the late-onset PPD [18]. Likewise, in a US-based cohort, Sheffield et al. discovered that increased PM_2.5_ exposure in mid-pregnancy was associated with higher anhedonia and depressive symptoms specifically in Black women [19]. He et al. found that pregnant women with no prior history of mental illness, exposed to noise, specifically night-time noise, have a higher risk of hospitalization for depression and other mental health disorders later in life [20]. In addition, Crockett et al. showed that the lack of public transportation acted as a barrier in accessing care for rural low-income African American pregnant women at risk for PPD [21]. Similarly, a study involving a home-based intervention for depression in low-income mothers noted that residing in neighborhoods with poor housing, higher crime rates, lack of essential resources increased the notion of uncertainty in life and participants required more encouragement to remain in the study [22].

 In this study, based on existing evidence above, we hypothesize that the built environment, through a wide range of measures influencing the accessibility to the transportation system and infrastructure elements, green space, and other urban structure, is associated with variability in prenatal care and subsequent maternal mental health outcomes. Given findings from previous literature on the impact of the built environment on women’s mental health and use of healthcare, we defined PPD as our primary outcome [24]. PPD has been associated with increased infant mortality, higher rates of hospitalizations, impaired mother-child attachment, developmental problems in children, and increased stress within families [25–28]. The plethora of physical and psychological effects of PPD reported in previous studies include postpartum weight retention, reduced physical health, bodily pain, anxiety, low self-esteem, risky addictive behavior of substances, and suicide ideation [29]. The biological risk factors of PPD include genetic factors, age, pregnancy complications, medical illness, and smoking during pregnancy [4, 30–32]. The social, cultural, and environmental risk factors include income status, domestic violence, lack of social support, quantity and quality of green spaces, and residential noise pollution [31, 33–37].

We tested our hypotheses by linking patients’ health data extracted from de-identified electronic health records (EHR) with publicly available census-tract level data on the built environment. Routinely collected from clinical encounters, EHR data capture detailed longitudinal health data on health and health service utilization. Increasingly, EHR data have been used as a source of longitudinal data in population health studies for its ability to provide detailed and rich health information within patient cohorts [38]. Leveraging a large cohort of nearly 9,000 women in New York City from 2015 to 2017, we applied machine learning algorithms to EHR data to identify patterns in prenatal care [39]. We then evaluated the relationships among prenatal care patterns, PPD incidence, and the built environment using structural equation modeling [40]. The association found may inform patients, care providers, and public health policymakers in supporting a healthy pregnancy and new motherhood through a better understanding of the built environment as a modifiable social determinant of health.

## Methods

### Study setting

#### EHR data

**Fig. 1:**
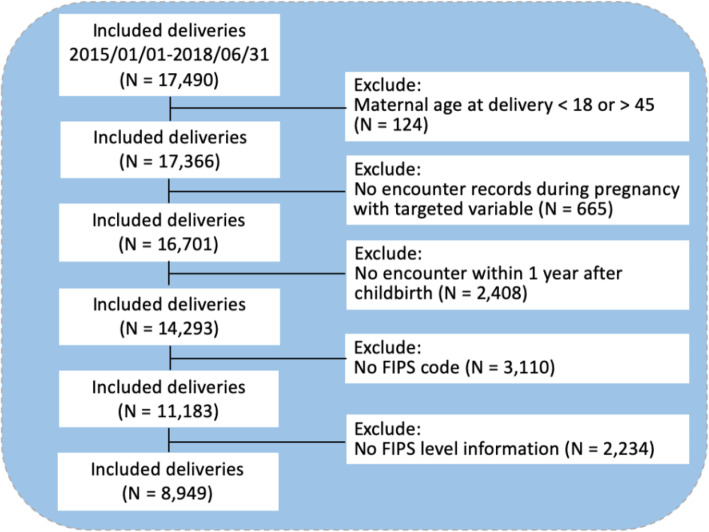
Study cohort inclusion and exclusion criteria

EHR data on 8,949 pregnant women from an urban academic medical center from 2015 to 2017 were extracted. The cohort inclusion and exclusion criteria are described in Fig. 1. We excluded patients whose ages were below 18 or above 45, had no encounter recorded in the EHR from 1 year prior to pregnancy to 1 year after delivery, or missing home locations information. We extracted patient information including gender, age, race, ethnicity, body mass index (BMI), marital status, outpatient and inpatient diagnoses, outpatient and inpatient prescription medication orders, and corresponding encounter dates from the EHR data. Patient age was calculated as the time difference between the birth date and first prenatal checkup date. The gestational week was calculated using the date of delivery and the specific gestational age at prenatal checkups. Marital status was defined as single (single, divorced, widowed, unknown), and married, as extracted from unstructured clinical notes using regular expression. The trimester of each event was determined using the difference in time between each event and delivery. All diagnoses were represented as Systematized Nomenclature of Medicine-Clinical Terms (SNOMED-CT) codes [41]. Anatomical Therapeutic Chemical (ATC) Classification System was used to standardize the specific drug prescription and dosage information [42]. The primary outcome of PPD was defined as having at least one diagnosis of depression within 1 year after childbirth based on SNOMED codes (see Additional file 1).

### Built environment data

#### Accessibility to public and active transportation, and green spaces

Three indicators were defined to measure the accessibility to public and active transportation facilities [43–46] within a 500-meter radius [47]: the number of bus stops, the number of subway stations within, and the length of bike paths within. The spatial data on public transportation and bike facilities were obtained in shapefile formats from New York State [48]. We used ArcGIS 10.6 spatial analysis tools to count the number of bus stops and subway stations within each 500-meter radius around each patients’ home location and also to measure the length of bike paths within the 500-meter radius. Access to green spaces, as defined by the City of New York under recreation land use, were calculated using the green areas that fall within the 500-meter buffer.

#### Exposure to traffic

We obtained traffic data from the New York activity-based travel demand model referred to as “New York Best Practice Model (NYBPM)” [49]. The model predicts daily traffic volume in each roadway link for the different types of vehicles by two categories: light- (passenger vehicles and taxis) and heavy-duty (buses and trucks) vehicles for their different levels of health impacts [50]. The vehicle kilometer traveled (VKT) as an indicator for travel activity within the 500-meter radius was then calculated. The buffer was chosen since both monitoring [51] and simulation [52] studies have shown that vehicle pollution concentration reaches the background level. VKT is calculated by multiplying traffic volume by the distance of travel, representing the amount of traffic activity.

#### Land use

Four indicators were defined to measure the role of land use: entropy-based land use mix (LUM) index, retail floor area ratio (RetFAR), street connectivity, and sidewalk availability. The variables measure the availability and variety of land use types (i.e., type of activity) within 500 m of the subject’s home location. The land use data including information about land use class and parcel area at the parcel level were extracted from the parcel shapefile obtained from New York State [48]. The LUM index within a 500-meter radius measures the heterogeneity of land use, such as residential, commercial, retail, and industrial, within the radius [53]. The LUM index ranges between 0 and 1, where 0 represents homogeneity and 1 represents maximum heterogeneity [53]. Higher LUM values indicate higher walkability of the area. The RetFAR is the retail building floor area divided by the retail land area within the 250-m radius [53]. RetFAR is indicative of pedestrian-orientated design and higher walkability. Examples with higher and lower RetFAR are multi-floor departmental stores and open-style outlets, respectively. The number of intersections within the 500-meter radius is another land use indicator used to measure the walkability of the neighborhood [54]. The number of intersections was extracted from the transportation network developed for the NYBPM travel demand model. To calculate the sidewalk area, as a measure of access to walking facilities, within the 500-meter radius, we used the sidewalk shapefiles [49].

#### Air pollution

PM_2.5_ and ozone (O_3_) concentrations at the census tract level for the period of 2015–2017 were obtained from the Center for Air, Climate and Energy Solutions which applied Land Use Regression (LUR) models to estimate every subject’s exposure to air pollution [55]. PM_2.5_ and O_3_ together could represent both regional background and hotspot air pollution levels.

#### Other SDoH

Lastly, SDoH information at the census-tract (11-digit Federal Information Processing Standard code) level was extracted using the FACETS dataset [56]. Variables used in the analysis included census-tract level average percent of college degree education, GINI index, and uninsured percentage from American Community Survey, a binary indicator of low access to healthy food within half-mile from the Food Access Research Atlas, United States Department of Agriculture, the population-weighted distance to closest 7 parks from the Centers for Disease Control and Prevention, and lastly walk score scales the from Rundle-Columbia Built Environment and Health Research Group.

### Patterns of prenatal care

We extracted the health and healthcare utilization information during the prenatal period for each patient from the EHR data. Patients who had similar overall prenatal care patterns were categorized into clusters as having experienced generally similar prenatal events. The similarity between pairs of patients was measured using the longest common subsequence (LCS) distance. LCS measures the longest overlap that 2 sequences have in common; thus, larger LCS indicates a more similar course of the clinical events. In this study, we compared the sequence of each patient’s clinical events (e.g., encounters, diagnoses, prescription medications) to others in the cohort to generate pairs of LCS distances. Based on the similarity, the categorization of patients was performed using the hierarchical clustering algorithm, a well-established machine learning method for detecting underlying clusters in a population [39]. The final number and size of the clusters were determined using the Silhouette value [39]. This method was previously used to mine EHR data to identify health and healthcare utilization patterns among patients with chronic kidney disease, heart failure, and undifferentiated abdominal pain [39, 57, 58]. Because of the large number (n > 6,000) of unique clinical events recorded in the EHR data, we limited the pattern mining to focus on variables that were found to be most predictive of PPD in a related work preparatory to this study [59]. The list of variables, including complications during pregnancy and medication usage, are shown in Additional file 2. The cluster analysis was done in Python 3.6.5 and R 4.0.0.

Within each cluster, we applied a well-established sequence mining algorithm, Sequential Pattern Discovery using Equivalent Classes (SPADE) algorithm, [60] to discover and visualize common patterns within each cluster identified above. These patterns include sequential events that are shared by a large enough portion of the patients. For the implementation of SPADE, we used the “arulesSequences package” in R version 3.4.3 (R Foundation for Statistical Computing, Vienna, Austria).

### Statistical analysis

The distribution of study variables described in sections EHR Data and Built Environment Data (Table 1) were assessed within each identified cluster. Multivariate Imputation by Chained Equations (MICE) was used to address the missing value issue [61]. We further studied the relationship between prenatal care, as reflected by the cluster membership, the built environment characteristics, and incidence of PPD using structural equation models (SEMs) [40]. Two SEMs were constructed for the primary and secondary outcomes separately. All independent variables were considered, but removed if there was multicollinearity as determined by variable inflation factor larger than 10. Statistical analysis was done using Stata/IC 16.0 and R 4.0.0. We applied Chi-square tests for categorical variables and analysis of variance (ANOVA) for continuous variables to compare the differences across clusters. *P*-value of 0.05 was used as the significance threshold.

**Table 1:** Descriptive statistics of the study cohort

Variables	Values
**Demographics**	
Age, mean (SD), year	33.69 (4.59)
Pre-pregnancy BMI, mean (SD), kg/m^2^	23.77 (4.31)
Gestational Week, mean (SD), week	38.69 (2.09)
Race, No. (%)	
White	4409 (49.27)
Asian	1689 (18.87)
Black or African American	560 (6.26)
Other	976 (10.91)
Unknown	1315 (14.69)
Marital Status, No. (%)	
Single	1193 (13.33)
Married	7756 (86.67)
Cesarean Section, No. (%)	
Yes	1878 (20.99)
No	7071 (79.01)
Insurance, No. (%)	
Commercial	7519 (84.02)
Medicaid	1226 (13.70)
Other	204 (2.28)
**Built Environment**	
Number of bus stops within 500 m radius, mean (SD)	25.26 (10.0)
Number of subway stations within 500 m radius, mean (SD)	1.81 (1.83)
Parks Area within 500 m radius, mean (SD), m^2^	463112.43 (660506.3)
Bike Path Length within 500 m radius, mean (SD), m	29070.94 (15172.89)
VKT of light vehicles within 500 m radius, mean (SD), 100,000 units	3283.87 (2242.98)
VKT of heavy vehicles within 500 m radius, mean (SD), 10,000 units	3608.43 (2516.02)
LUM index within 500 m radius, mean (SD)	0.64 (0.17)
RetFar within 500 m radius, mean (SD)	0.24 (0.23)
Number of Intersections within 500 m radius, mean (SD)	12.06 (7.76)
Sidewalk Area within 500 m radius, mean (SD), 1000 m^2^	907.77 (208.53)
Ozone Concentration, mean (SD), µg/m^3^	46.56 (0.50)
PM_2.5_ Concentration, mean (SD), µg/m^3^	9.28 (0.47)
Percent of Colleges Degree, mean (SD), %	35.79 (11.49)
Average Poverty Rate, mean (SD), %	1.62 (2.15)
Average Respiratory Hazard Index, mean (SD)	4.51 (1.16)
Low Access to Healthy Food, No. (%)	297 (3.32)
Uninsured Percentage, mean (SD), %	8.26 (5.60)
**Postpartum Depression**	
Yes, No. (%)	273 (3.05)
**Average number of ED visits per patient**	
Pre-delivery (*N* = 3900, 43.58 %), mean (SD)	0.74 (1.16)
Post-delivery (*N* = 482, 5.39 %), mean (SD)	0.07 (0.31)

## Results

Table 1 shows the descriptive statistics of the study cohort where continuous variables are presented as mean (standard deviation (SD)), and categorical variables are presented as N (% in total cohort). The average age of our patient population was 33.7 years (SD = 4.59). Nearly half (49.27 %) of the patients were White, and the majority were married (86.7 %) and had Commercial insurances (84.0 %). Over 3 % of the cohort were diagnosed with PPD. A total of 3,900 (43.6 %) and 482 (5.4 %) patients had at least one ED visit pre- and post-delivery.

We identified 3 clusters with 1,934 (cluster 1), 4,129 (cluster 2), and 2,886 (cluster 3) patients, respectively, based on their clinical event sequences. For the primary outcome of PPD, 6.72 % of the women in cluster 1 had a diagnosis of PPD within 1 year after childbirth, which was higher than clusters 2 (2.66 %) and 3 (1.14 %) (*P* < .001). Table 2 displays the distributions of variables used to determine the clusters. The distributions of variables were all statistically different across clusters except for antidepressant prescriptions and the diastolic blood pressure in the third trimester. Table 3 presents a post-hoc analysis of the distribution of demographics, medications, diagnoses, and built environment factors that were significantly different across the three clusters. The mean (SD) age across three clusters were 35.01 (4.73) years, 33.78 (4.29) years and 32.68 (4.66) years, respectively (*P* < .001). There were more unmarried patients in cluster 1 than the other two clusters (*P* < .001). In addition, the number of ED visits in both the pre- and post-delivery periods in cluster 1 was higher (*P* < .001) than the other clusters. We observed higher rates of prescription medications in cluster 1, such as analgesics, antipyretics and opioids (*P* < .001). Also, more patients in cluster 1 had complications during pregnancy, unplanned pregnancies, high-risk pregnancy, abnormal glucose level, elderly primigravida and advanced maternal age gravidas than the other two clusters (*P* < .001).

**Table 2:** Distribution of clinical pathway elements across clusters

Variables	Cluster 1 (*N* = 1934)	Cluster 2 (*N* = 4129)	Cluster 3 (*N* = 2886)	*P*-value
**Diagnosis**				
Anxiety history, no. (%)	111 (5.74)	86 (2.08)	26 (0.90)	< 0.001
Other disorder history, no. (%)	83 (4.29)	89 (2.16)	35 (1.21)	< 0.001
Mood disorder history, no. (%)	75 (3.88)	61 (1.48)	32 (1.11)	< 0.001
Depression in pregnancy, no. (%)	28 (1.45)	24 (0.58)	12 (0.42)	< 0.001
Anxiety in pregnancy, no. (%)	41 (2.12)	24 (0.58)	9 (0.31)	< 0.001
Mental disorder in pregnancy, no. (%)	21 (1.09)	22 (0.53)	13 (0.45)	0.014
Palpitations, no. (%)	57 (2.95)	56 (1.36)	19 (0.66)	< 0.001
Diarrhea, no. (%)	48 (2.48)	52 (1.26)	27 (0.94)	< 0.001
Vomiting in pregnancy, no. (%)	50 (2.59)	85 (2.06)	44 (1.52)	0.034
Hypertensive disorder, no. (%)	28 (1.45)	43 (1.04)	12 (0.42)	0.001
Acute pharyngitis, no. (%)	40 (2.07)	31 (0.75)	12 (0.42)	< 0.001
Hemorrhage in early pregnancy antepartum, no. (%)	29 (1.50)	35 (0.85)	15 (0.52)	0.002
Threatened miscarriage, no. (%)	170 (8.79)	164 (3.97)	54 (1.87)	< 0.001
Abdominal pain, no. (%)	195 (10.08)	241 (5.84)	112 (3.88)	< 0.001
Migraine, no. (%)	28 (1.45)	25 (0.61)	8 (0.28)	< 0.001
Hypothyroidism, no. (%)	337 (17.43)	342 (8.28)	148 (5.13)	< 0.001
Placental infarct, no. (%)	77 (3.98)	82 (1.99)	71 (2.46)	< 0.001
Deliveries by cesarean, no. (%)	510 (26.37)	833 (20.17)	535 (18.54)	< 0.001
Primigravida, no. (%)	1206 (62.36)	2453 (59.41)	1024 (35.48)	< 0.001
Pre-eclampsia, no. (%)	23 (1.19)	25 (0.61)	13 (0.45)	0.007
Abnormality of organs and/or soft tissues of pelvis affecting pregnancy, no. (%)	169 (8.74)	225 (5.45)	103 (3.57)	< 0.001
False labor at or after 37 completed weeks of gestation, no. (%)	31 (1.60)	93 (2.25)	101 (3.50)	< 0.001
**Medications**				
Antidepressants, no. (%)	12 (0.62)	14 (0.34)	6 (0.21)	0.061
Beta blocking agents, no. (%)	55 (2.84)	53 (1.28)	33 (1.14)	< 0.001
Antihistamines for systemic use, no. (%)	185 (9.57)	234 (5.67)	83 (2.88)	< 0.001
Direct acting antivirals, no. (%)	143 (7.39)	187 (4.53)	70 (2.43)	< 0.001
Other antibacterials, no. (%)	119 (6.15)	205 (4.96)	54 (1.87)	< 0.001
**Health Services**				
Pre-delivery ED visits (within 1-year), mean (SD)	1.12 (1.54)	0.68 (1.01)	0.56 (0.97)	< 0.001
**Vitals**				
Diastolic blood pressure in the third trimester	69.35 (6.22)	69.28 (5.86)	69.12 (4.76)	0.321
**Marital Status**				
Single (vs. Married), no. (%)	348 (17.99)	578 (14.0)	267 (9.25)	< 0.001
**Race , no. (%)**				
Asian	280 (14.48)	679 (16.44)	730 (25.29)	< 0.001
Black	145 (7.50)	260 (6.30)	155 (5.37)	
Other	229 (11.84)	477 (11.55)	270 (9.36)	
Unknown	202 (10.44)	564 (13.66)	549 (19.02)	
White	1078 (55.74)	2149 (52.05)	1182 (40.96)

**Table 3:** Post-hoc analysis of other demographic and clinical characteristics across clusters

Variables	Cluster			*P*-value
	1 (*N* = 1934)	2 (*N* = 4129)	3 (*N* = 2886)	
**Sociodemographic**				
Age, mean (SD), year	35.01 (4.73)	33.78 (4.29)	32.68 (4.66)	< 0.001
Average Poverty Rate, mean (SD), %	1.35 (1.83)	1.42 (1.87)	2.07 (2.61)	< 0.001
Cesarean Section, no. (%)				
Yes	510 (26.37)	833 (20.17)	535 (18.54)	< 0.001
No	1424 (73.63)	3296 (79.83)	2351 (81.46)	
Insurance, no. (%)				
Commercial	1603 (82.89)	3492 (84.57)	2424 (83.99)	0.45
Medicaid	283 (14.63)	552 (13.37)	391 (13.55)	
Other (Medicare, Self-pay, Unknown)	48 (2.48)	85 (2.06)	71 (2.46)	
**Other Medication Prescriptions**				
Other Analgesics and Antipyretics, no. (%)	324 (16.75)	534 (12.93)	324 (11.23)	< 0.001
Opioids, no. (%)	285 (14.74)	323 (7.82)	243 (8.42)	< 0.001
Thyroid Preparations, no. (%)	291 (15.05)	273 (6.61)	84 (2.91)	< 0.001
Drugs for Functional Gastrointestinal Disorders, no. (%)	171 (8.84)	235 (5.69)	150 (5.2)	< 0.001
Antiemetics and Antinauseants, no. (%)	170 (8.79)	242 (5.86)	145 (5.02)	< 0.001
Other Plain Vitamin Preparations, no. (%)	172 (8.89)	252 (6.10)	83 (2.88)	< 0.001
Beta-lactam Antibacterials, Penicillins, no. (%)	175 (9.05)	245 (5.93)	81 (2.81)	< 0.001
Progestogens, no. (%)	284 (14.68)	156 (3.78)	42 (1.46)	< 0.001
**Other Clinical Characteristics**				
Normal Delivery, no. (%)	1435 (74.2)	3346 (81.04)	2310 (80.04)	< 0.001
Complication Occurring During Pregnancy, no. (%)	887 (45.86)	1439 (34.85)	605 (20.96)	< 0.001
Unplanned Pregnancy, no. (%)	641 (33.14)	1178 (28.53)	742 (25.71)	< 0.001
Post-term Pregnancy, no. (%)	465 (24.04)	1116 (27.03)	532 (18.43)	< 0.001
Elderly Primigravida, no. (%)	674 (34.85)	935 (22.64)	360 (12.47)	< 0.001
High Risk Pregnancy, no. (%)	536 (27.71)	662 (16.03)	297 (10.29)	< 0.001
Abnormal Glucose Level, no. (%)	479 (24.77)	757 (18.33)	163 (5.65)	< 0.001
Advanced Maternal Age Gravida, no. (%)	416 (21.51)	675 (16.35)	222 (7.69)	< 0.001
Disorder of Pregnancy, no. (%)	342 (17.68)	499 (12.09)	276 (9.56)	< 0.001
Pre-pregnancy BMI, mean (SD), kg/m^2^	24.24 (5.19)	23.55 (4.32)	23.77 (3.54)	< 0.001
Gestational Week, mean (SD), week	38.58 (2.12)	38.83 (1.92)	38.55 (2.26)	< 0.001
Post-delivery ED visits (within 6-months), mean (SD)	0.10 (0.37)	0.06 (0.29)	0.05 (0.28)	< 0.001
**Postpartum Depression**				
Yes, no. (%)	130 (6.72)	110 (2.66)	33 (1.14)	< 0.001
No, no. (%)	1804 (93.28)	4019 (97.34)	2853 (98.86)	

**Fig. 2:**
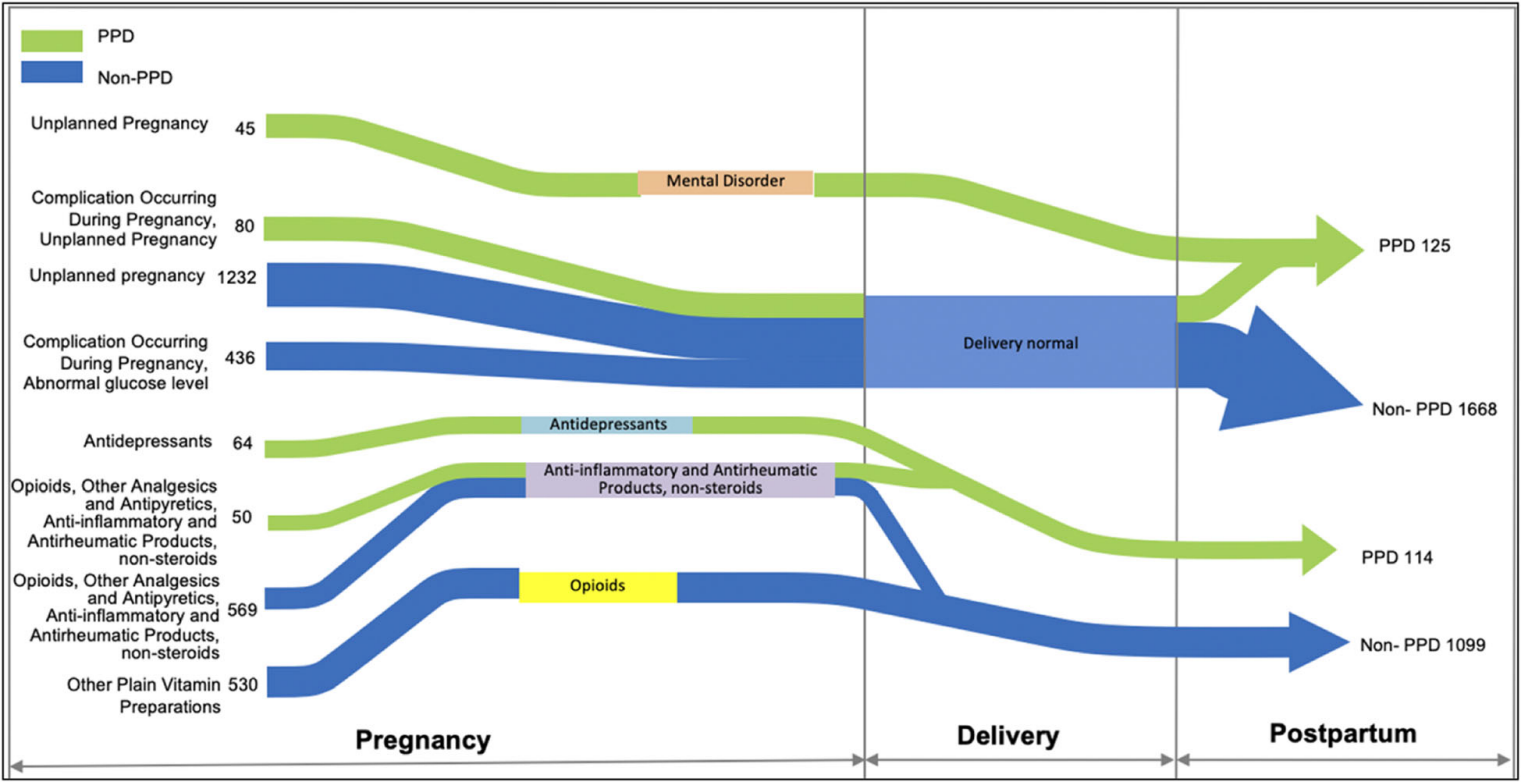
Selected patterns in prenatal care identified from the EHR

Figure 2 showcases sequential patterns in the prenatal care identified from the study data. Green pathways signify pathways leading to PPD diagnoses, in contrast to blue pathways that do not. The pathways are made up of events (left-hand side) which converge or diverge to other events (right-hand side) before the development, or the absence, of PPD. For example, we found that 45 mothers with PPD had unplanned pregnancies followed by diagnoses of mental health disorders during pregnancy. Also, we observed that 64 mothers who developed PPD had multiple prescriptions of antidepressants during pregnancy. Another pattern indicated 50 mothers who developed, and 569 mothers who did not develop, PPD were prescribed Opioids, Other Analgesics and Antipyretics, and Anti-inflammatory and Antirheumatic Products, non-steroids, before the refill of Anti-inflammatory and Antirheumatic Products, non-steroids.

Table 4 displays the results from the SEM for the outcome of PPD. Regarding the primary outcome, patients in clusters 1 (odds ratio = 6.3, *P* < .001) and 2 (odds ratio = 2.43, *P* < .001) are more likely to have a diagnosis PPD within 12 months after childbirth than women in cluster 3. Relative to cluster 3, patients in cluster 1 are more likely to have patients living in census tracts that have lower PM 2.5 (odds ratio = 0.858, *P* = .02), lower retail floor area ratio (odds ratio = 0.882, *P* = .03), lower LUM (odds ratio = 0.508, *P* < .001), higher GINI (odds ratio = 4.317, *P* = .002), and higher college degree percentage (odds ratio = 4.401, *P* < .001). Patients are also more likely to be older (odds ratio = 1.115, *P* < .001) and not married (odds ratio = 0.404, *P* < .001). Relative to cluster 3, patients in cluster 2 are more likely to have patients living in census tracts that have lower PM 2.5 (odds ratio = 0.890, *P* = .03), lower retail floor area ratio (odds ratio = 0.867, *P* = .001), lower GINI (odds ratio = 0.412, *P* = .02), and higher college degree percentage (odds ratio = 4.996, *P* < .001). Patients are also moderately more likely to be older (odds ratio = 1.046, *P* < .001) and not married (odds ratio = 0.560, *P* < .001). Race and insurance types (commercial, Medicaid, Other including Medicare) were not significantly associated with the cluster membership in the models although the unadjusted association was significant.

**Table 4:** Structural equation model results. OR: odds ratio

	Variable	OR	*P*-value
PPD	Cluster 1	6.3	<.001
	Cluster 2	2.43	<.001
Cluster 1 (vs. Cluster 3 as reference)	Retail	0.882	.03
	PM_2.5_	0.858	.02
	Age	1.115	<.001
	Married	0.404	<.001
	LUM	0.508	<.001
	GINI	4.317	.002
	College	4.401	<.001
Cluster 2 (vs. Cluster 3 as reference)	Retail	0.867	.001
	PM_2.5_	0.890	.03
	Age	1.046	<.001
	Married	0.560	<.001
	LUM	0.749	.06
	GINI	0.412	.02
	College	4.996	<.001

*PPD* postpartum depression, *PM*_*2.5*_ particulate matter ≤ 2.5 μm in diameter, *LUM* land use mix, *GINI* GINI inequality index

 We further contrasted the characteristics of PPD cases across clusters as shown in Additional file 3. The association between PPD and the built environment factors was examined and shown in Additional file 4. The factors that were significantly associated with increased risk for PPD were the number of intersections within a 500-meter radius, the number of bus stops within a 500-meter radius, and retail floor area ratio, while adjusting for GINI index for income inequality which were also significant in the model.

## Discussion

There were two major findings in this study. Three clusters of prenatal health and healthcare utilization patterns were discovered from a cohort of women whose pregnancies were managed entirely or partially in an urban academic medical center from 2015 to 2017. The distribution of the primary outcome, PPD, was significantly different across the clusters. Clinically, the clusters differed in maternal age, BMI, marital status, medication use, chronic conditions, and complications during pregnancy. In addition, we found that the cluster membership was associated with built environment factors related to walkability, access to retail resources, air quality, and neighborhood income equality. These findings contribute to the growing body of evidence that the built environment in the community confers an impact on the trajectories of health and health service utilization during pregnancy.

The associations found between retail, land use and the study outcomes among the pregnant cohort are novel and important contributions to the literature. The mixed land use and more retail access may be a proxy for the connectedness of the neighborhood in providing community support to women. These community resources potentially lead to increased opportunities for social contact, lower stress levels, and higher physical activity levels, which is consistent with previous literature tying maternal mental health to green space [9, 10]. Air quality has been linked with adverse birth outcomes including preterm birth and miscarriages in previous literature [9]. However, we found that lower PM_2.5_ concentration to be associated with clusters with higher PPD incidences in contrary to previous literature. In our urban study setting, PM_2.5_ concentration is highest in the most affluent area and becomes lower as we move out to other parts of the study setting. Therefore, our findings on the association of poor air quality with higher incidence of PPD potentially reflect patient cohorts who are predominantly in or outside the most affluent part of the city who have better access to mental health reporting and care. Patterns learned from this study may inform expecting and new mothers, their care providers, as well as guideline and policymakers, to better prepare and navigate pregnancy and postpartum care. Additionally, our findings may have implications for policies during the current COVID-19 pandemic as our communities and their stores face significant changes.

There are limitations to the study. All diagnoses in the study were defined using diagnostic codes. Therefore, missed and under-diagnosis of health conditions during pregnancy, including PPD, is a crucial limitation. It is possible that this study missed PPD patients who did not disclose symptoms due to stigma against mental health, and patients who were diagnosed outside of our health system. The underdiagnosis and misdiagnosis may be more prevalent among women who live in low-income neighborhoods. Some of these limitations may be addressed in future work by patient interviews and questionnaires, and prospective cohort studies. Additionally, the application of natural language processing on unstructured clinical notes may allow us to elicit underdiagnosed and missed PPD as well as other conditions. Moreover, we were not able to address the possible reporting bias in our study population with respect to information such as race and marital status. Nearly 15 % of the racial information was unknown from the EHR data. Future studies may explore the leveraging of patient-reported outcome data in overcoming this limitation. Furthermore, in analyzing the medication data, we did not consider the dose-response relationship between medications and the outcome as prescription fill information was not available. Detailed medication dose and frequency information can be analyzed in future work if pharmacy claims data become available. PPD cases across the clusters did not differ significantly in our analysis, but this may be due to the small sample size. Applying our methods to a larger patient cohort may allow us to further delineate the association of the built environment and potentially different PPD patient types. Lastly, while this study used data from a single health system in NYC, further work will aim to validate our findings using EHR data from other institutions and across different cities in the US.

## Conclusions

We found that the built environment quality is associated with variability in prenatal care and maternal mental health outcomes in a large retrospective cohort study using EHR data. Built environment qualities that were identified in a structural equation model include LUM and RetFAR as indicators for walkability and street connectivity, and air quality. Findings from this study may inform healthcare providers and public health policymakers in understanding modifiable risk factors such as isolation that are associated with poor pregnancy care and outcomes.

## Supplementary information

Additional file 1. Definition of PPD based on SNOMED codes

Additional file 2. Variables used in the construction of the clinical pathways

Additional file 3. Characteristics of PPD cases across clusters

Additional file 4. Associations between PPD and the built environment variables in the study cohort

## Data Availability

The datasets generated and/or analyzed during the current study are not publicly available due to its inclusion of patient health information protected by the Health Insurance Portability and Accountability Act but are available from the corresponding author on reasonable request. Analysis was conducted in StataIC 16.1.

## Abbreviations

SDoH: social determinants of health
ED: emergency departments
PPD: postpartum depression
PM_2.5_: particulate matter ≤ 2.5 μm in diameter
EHR: electronic health records
BMI: body mass index
SNOMED-CT: Systematized Nomenclature of Medicine-Clinical Terms
ATC: Anatomical Therapeutic Chemical
NYBPM: New York Best Practice Model
VKT: vehicle kilometer traveled
LUM: land use mix
RetFAR: retail floor area ratio
O_3_: ozone
LUR: Land Use Regression
LCS: longest common subsequence
SPADE: Sequential Pattern Discovery using Equivalent Classes
MICE: Multivariate Imputation by Chained Equations
SEM: structural equation model
ANOVA: analysis of variance
SD: standard deviation
