# Supplementary material for: Identifying urban built environment factors in pregnancy care and maternal mental health outcomes
Source: BMC Pregnancy Childbirth. 2021 Sep 4;21:599. doi: 10.1186/s12884-021-04056-1 (PMC8417675; doi:10.1186/s12884-021-04056-1)
Supplement: Supplementary file 1 — Definition of PPD based on SNOMED codes [file 12884_2021_4056_MOESM1_ESM.docx]

**Identifying Urban Built Environment Factors in Pregnancy Care and Maternal Mental Health Outcomes**

Yiye Zhang, PhD^1,2^; Mohammad Tayarani, PhD^3^; Shuojia Wang, PhD^4^; Yifan Liu, MS^1^; Mohit Sharma, MS^1^; Rochelle Joly, MD^5^; Arindam RoyChoudhury, PhD^1^, Alison Hermann, MD^6^; Oliver H. Gao, PhD^7^; Jyotishman Pathak, PhD^1,6^

1. Department of Population Health Sciences, Weill Cornell Medicine, New York, NY, USA

2. Department of Emergency Medicine, Weill Cornell Medicine, New York, NY, USA

3. School of Civil and Environmental Engineering, Cornell University, Ithaca, NY, USA

4. Tencent Jarvis Lab, Shenzhen Guangdong, China

5. Department of Obstetrics and Gynecology, Weill Cornell Medicine, New York, NY, USA

6. Department of Psychiatry, Weill Cornell Medicine, New York, NY, USA

Corresponding author: Yiye Zhang, PhD, MS, 425 East 61st Street, New York, NY 10065, yiz2014@med.cornell.edu, (646) 962-9437

**Additional file 1. Definition of PPD based on SNOMED codes**

| **Concept name** | **SNOMED code** |
| --- | --- |
| Acute depression | 712823008 |
| Adjustment disorder with depressed mood | 57194009 |
| Adjustment disorder with depressed mood in remission | 698696007 |
| Adjustment disorder with mixed anxiety and depressed mood | 782501005 |
| Anxiety | 48694002/ 38237000 |
| Anxiety disorder | 197480006/ 191703000/ 65673007 |
| Anxiety disorder in mother complicating childbirth | 10743001000119100 |
| Anxiety in pregnancy | 94641000119109 |
| Anxiety state | 198288003/154882009 |
| Anxiety state NOS | 191711005 |
| Anxiety states | 268752000 |
| Chronic anxiety | 191708009 |
| Depressed mood | 366979004/41006004/367204005 |
| Depressed mood with postpartum onset | 704678007 |
| Depression - postnatal | 154889000 |
| Depression NOS | 307537002/154963001 |
| Depressive conduct disorder | 192605002 |
| Depressive disorder in mother complicating pregnancy | 94631000119100 |
| Depressive disorder in remission | 698957003 |
| Generalized anxiety disorder | 21897009/191706008/192401002 |
| Major depression in full remission | 63412003 |
| Major depression in partial remission | 30605009 |
| Major depression in remission | 42810003 |
| Major depression single episode, in partial remission | 70747007 |
| Major depression, single episode | 36923009 |
| Major depression, melancholic type | 320751009/62951006 |
| Major depressive disorder | 370143000 |
| Major depressive disorder, single episode with atypical features | 42925002 |
| Major depressive disorder, single episode with melancholic features | 63778009 |
| Major depressive disorder, single episode with postpartum onset | 25922000 |
| Mild anxiety | 70997004 |
| Mild depression | 310495003/154965008/390717003 |
| Mild postnatal depression | 237349002 |
| Mild major depression | 87512008 |
| Minimal depression | 718636001 |
| Minimal major depression | 720455008 |
| Minimal major depression single episode | 720454007 |
| Minimal recurrent major depression | 720451004 |
| Minor depressive disorder | 48589009 |
| Mixed anxiety and depressive disorder | 231504006/154964007/191707004/231504006/154964007/191707004 |
| Postpartum depression | 58703003 |
| Perinatal depression | 10211000132109 |
| Postnatal depression | 191740008 |
| Postnatal depressive disorder | 147016002 |
| Mild postnatal depression | 237349002 |
| Moderate anxiety | 61387006 |
| Moderate depression | 310496002/154919005/154966009 |
| Moderate major depression | 832007 |
| Moderately severe depression | 719593009 |
| Moderately severe major depression | 719592004 |
| Moderately severe major depression single episode | 720453001 |
| Moderately severe recurrent major depression | 720452006 |
| Mood disorder with major depressive-like episode due to general medical condition | 77486005 |
| Severe major depression | 450714000 |
| Severe depression | 310497006/154967000 |
| Severe postnatal depression | 237350002 |
| Severe recurrent major depression | 281000119103 |
| Severe major depression, single episode | 251000119105 |
| Severe major depression without psychotic features | 75084000 |
| Severe recurrent major depression without psychotic features | 36474008 |
| Severe anxiety | 80583007 |
| Single episode of major depression in full remission | 19527009 |
| Single major depressive episode | 268620009/192366006/ |
| Single major depressive episode, in full remission | 191606003 |
| Single major depressive episode, in partial or unspecified remission | 191605004 |
| Single major depressive episode, mild | 191601008 |
| Single major depressive episode, moderate | 191602001 |
| Single major depressive episode, unspecified | 191600009 |
| Recurrent anxiety | 191709001 |
| Recurrent major depression | 66344007 |
| Recurrent major depression in full remission | 46244001 |
| Recurrent major depression in remission | 68019004 |
| Recurrent major depressive disorder with postpartum onset | 71336009 |
| Recurrent major depressive disorder with atypical features | 38694004 |
| Recurrent major depressive episode NOS | 191617002 |
| Recurrent major depressive episodes, in full remission | 191615005 |
| Recurrent major depressive episodes, in partial or unspecified remission | 191614009 |
| Recurrent major depressive episodes, unspecified | 191609005 |
| Acute depression | 712823008 |
| Adjustment disorder with depressed mood | 57194009 |
| Adjustment disorder with mixed anxiety and depressed mood | 782501005 |
