# Supplementary material for: Identifying urban built environment factors in pregnancy care and maternal mental health outcomes
Source: BMC Pregnancy Childbirth. 2021 Sep 4;21:599. doi: 10.1186/s12884-021-04056-1 (PMC8417675; doi:10.1186/s12884-021-04056-1)
Supplement: Supplementary file 2 — Variables used in the construction of the clinical pathways [file 12884_2021_4056_MOESM2_ESM.docx]

**Identifying Urban Built Environment Factors in Pregnancy Care and Maternal Mental Health Outcomes**

Yiye Zhang, PhD^1,2^; Mohammad Tayarani, PhD^3^; Shuojia Wang, PhD^4^; Yifan Liu, MS^1^; Mohit Sharma, MS^1^; Rochelle Joly, MD^5^; Arindam RoyChoudhury, PhD^1^, Alison Hermann, MD^6^; Oliver H. Gao, PhD^7^; Jyotishman Pathak, PhD^1,6^

1. Department of Population Health Sciences, Weill Cornell Medicine, New York, NY, USA

2. Department of Emergency Medicine, Weill Cornell Medicine, New York, NY, USA

3. School of Civil and Environmental Engineering, Cornell University, Ithaca, NY, USA

4. Tencent Jarvis Lab, Shenzhen Guangdong, China

5. Department of Obstetrics and Gynecology, Weill Cornell Medicine, New York, NY, USA

6. Department of Psychiatry, Weill Cornell Medicine, New York, NY, USA

Corresponding author: Yiye Zhang, PhD, MS, 425 East 61st Street, New York, NY 10065, yiz2014@med.cornell.edu, (646) 962-9437

**Additional file 2. Variables used in the construction of the clinical pathways**

| **Variables** |
| --- |
| Anxiety history |
| Other disorder history |
| Antidepressants |
| Mood disorder history |
| Depression in pregnancy |
| Anxiety in pregnancy |
| Mental disorder in pregnancy |
| Palpitations |
| Diarrhea |
| Vomiting in pregnancy |
| Hypertensive disorder |
| Acute pharyngitis |
| Hemorrhage in early pregnancy antepartum |
| Threatened miscarriage |
| Abdominal pain |
| Migraine |
| Beta blocking agents |
| Antihistamines for systemic use |
| Hypothyroidism |
| Placental infarct |
| Single (vs. Married) |
| Deliveries by cesarean |
| Direct acting antivirals |
| Primigravida |
| Pre-eclampsia |
| Other antibacterials |
| ED visit |
| Abnormality of organs and/or soft tissues of pelvis affecting pregnancy |
| ﻿Diastolic blood pressure in the third trimester |
| False labor at or after 37 completed weeks of gestation |
| Race |
