# Supplementary material for: Identifying urban built environment factors in pregnancy care and maternal mental health outcomes
Source: BMC Pregnancy Childbirth. 2021 Sep 4;21:599. doi: 10.1186/s12884-021-04056-1 (PMC8417675; doi:10.1186/s12884-021-04056-1)
Supplement: Supplementary file 3 — Characteristics of PPD cases across clusters [file 12884_2021_4056_MOESM3_ESM.docx]

**Identifying Urban Built Environment Factors in Pregnancy Care and Maternal Mental Health Outcomes**

Yiye Zhang, PhD^1,2^; Mohammad Tayarani, PhD^3^; Shuojia Wang, PhD^4^; Yifan Liu, MS^1^; Mohit Sharma, MS^1^; Rochelle Joly, MD^5^; Arindam RoyChoudhury, PhD^1^, Alison Hermann, MD^6^; Oliver H. Gao, PhD^7^; Jyotishman Pathak, PhD^1,6^

1. Department of Population Health Sciences, Weill Cornell Medicine, New York, NY, USA

2. Department of Emergency Medicine, Weill Cornell Medicine, New York, NY, USA

3. School of Civil and Environmental Engineering, Cornell University, Ithaca, NY, USA

4. Tencent Jarvis Lab, Shenzhen Guangdong, China

5. Department of Obstetrics and Gynecology, Weill Cornell Medicine, New York, NY, USA

6. Department of Psychiatry, Weill Cornell Medicine, New York, NY, USA

Corresponding author: Yiye Zhang, PhD, MS, 425 East 61st Street, New York, NY 10065, yiz2014@med.cornell.edu, (646) 962-9437

**Additional file 3. Characteristics of PPD cases across clusters**

| **Variables (PPD=1)** | **Cluster by PPD Risk** | | |
| --- | --- | --- | --- |
|  | **High**  **(N=1934,**  **6.72% PPD)** | **Moderate**  **(N=4129,**  **2.66% PPD)** | **Low**  **(N=2886,**  **1.14% PPD)** |
| **Demographics** |  |  |  |
| Age, mean (SD), year | 34.68 (4.34) | 33.85 (4.57) | 34.82 (4.57) |
| Pre-pregnancy BMI, mean (SD), kg/m^2^ | 24.11 (5.78) | 22.71 (3.40) | 24.68 (3.87) |
| Gestational Week, mean (SD), week | 38.38 (2.74) | 38.39 (2.42) | 38.39 (3.61) |
| Race, No. (%) |  |  |  |
| White | 77 (59.23) | 66 (60.0) | 18 (54.55) |
| Asian | 14 (10.77) | 13 (11.82) | 2 (6.06) |
| Black or African American | 4 (3.08) | 7 (6.36) | 2 (6.06) |
| Other | 16 (12.31) | 15 (13.64) | 4 (12.12) |
| Unknown | 19 (14.62) | 9 (8.18) | 7 (21.21) |
| Marital Status, No. (%) |  |  |  |
| Single (vs. Married) | 27 (20.77) | 19 (17.27) | 7 (21.21) |
| **ED Visits** |  |  |  |
| Pre-delivery, mean (SD) | 1.17 (1.49) | 0.95 (1.27) | 0.76 (0.75) |
| Post-delivery, mean (SD) | 0.15 (0.40) | 0.08 (0.28) | 0.12 (0.42) |
| **Cesarean Section** |  |  |  |
| Yes, No. (%) | 39 (30.0) | 27 (24.55) | 9 (27.27) |
