# Supplementary material for: Identifying urban built environment factors in pregnancy care and maternal mental health outcomes
Source: BMC Pregnancy Childbirth. 2021 Sep 4;21:599. doi: 10.1186/s12884-021-04056-1 (PMC8417675; doi:10.1186/s12884-021-04056-1)
Supplement: Supplementary file 4 — Associations between PPD and the built environment variables in the study cohort [file 12884_2021_4056_MOESM4_ESM.docx]

**Identifying Urban Built Environment Factors in Pregnancy Care and Maternal Mental Health Outcomes**

Yiye Zhang, PhD^1,2^; Mohammad Tayarani, PhD^3^; Shuojia Wang, PhD^4^; Yifan Liu, MS^1^; Mohit Sharma, MS^1^; Rochelle Joly, MD^5^; Arindam RoyChoudhury, PhD^1^, Alison Hermann, MD^6^; Oliver H. Gao, PhD^7^; Jyotishman Pathak, PhD^1,6^

1. Department of Population Health Sciences, Weill Cornell Medicine, New York, NY, USA

2. Department of Emergency Medicine, Weill Cornell Medicine, New York, NY, USA

3. School of Civil and Environmental Engineering, Cornell University, Ithaca, NY, USA

4. Tencent Jarvis Lab, Shenzhen Guangdong, China

5. Department of Obstetrics and Gynecology, Weill Cornell Medicine, New York, NY, USA

6. Department of Psychiatry, Weill Cornell Medicine, New York, NY, USA

Corresponding author: Yiye Zhang, PhD, MS, 425 East 61st Street, New York, NY 10065, yiz2014@med.cornell.edu, (646) 962-9437

**Additional file 4**. Associations between PPD and the built environment variables in the study cohort

| **Variables** | **PPD** | **non-PPD** | **P-value** |
| --- | --- | --- | --- |
| Number of bus stops within 500 m radius, mean (SD) | 26.51 (10.12) | 25.22 (10.0) | .04* |
| Number of subway stations within 500 m radius, mean (SD) | 1.88 (1.79) | 1.81 (1.83) | .51 |
| Parks Area within 500 m radius, mean (SD), m^2^ | 433147.66 (660147.75) | 464055.30 (660533.55) | .45 |
| Bike Path Length within 500 m radius, mean (SD), m | 30037.96 (14528.24) | 29040.51 (15192.53) | .29 |
| VKT of light vehicles within 500 m radius, mean (SD), 100,000 units | 3179.51 (2335.33) | 3287.15 (2240.08) | .44 |
| VKT of heavy vehicles within 500 m radius, mean (SD), 10,000 units | 3799.90 (2606.12) | 3602.41 (2513.05) | .20 |
| LUM index within 500 m radius, mean (SD) | 0.64 (0.18) | 0.64 (0.17) | .78 |
| RetFar within 500 m radius, mean (SD) | 0.25 (0.21) | 0.24 (0.23) | .38 |
| Number of Intersections within 500 m radius, mean (SD) | 13.20 (8.50) | 12.03 (7.74) | .03* |
| Sidewalk Area within 500 m radius, mean (SD), 1000 m^2^ | 915.62 (224.85) | 907.52 (208.01) | .53 |
| Ozone Concentration, mean (SD), μg/m^3^ | 46.53 (0.44) | 46.56 (0.50) | .28 |
| PM_2.5_ Concentration, mean (SD), μg/m^3^ | 9.25 (0.46) | 9.28 (0.47) | .25 |
